# Supplementary material for: Reference library for suspect screening of environmental toxicants using ion mobility spectrometry-mass spectrometry
Source: Commun Chem. 2025 Aug 1;8:224. doi: 10.1038/s42004-025-01619-7 (PMC12317010; doi:10.1038/s42004-025-01619-7)
Supplement: Supplementary file 1 — Supplementary Information [file 42004_2025_1619_MOESM1_ESM.pdf]

Supporting Information File:

Reference Library for Suspect Screening of Environmental Toxicants Using Ion Mobility Spectrometry-Mass Spectrometry

Devin Teri[1], Noor A. Aly[1], James N. Dodds[4], Antony J. Williams[2], Jian Zhang[3], Paul A. Thiessen[3], Evan E. Bolton[3], Kara M. Joseph[4], Emma l. Schymanski[5], Ivan Rusyn[1]*, Erin S. Baker[4]*

[1]Department of Veterinary Physiology and Pharmacology, Texas A&M University, College Station, Texas 77843, USA

[2]Center for Computational Toxicology and Exposure, Office of Research and Development, U.S. Environmental Protection Agency, Research Triangle Park, North Carolina 27711, USA

[3]National Center for Biotechnology Information, National Library of Medicine, National Institutes of Health, Bethesda, MD 20894, USA

[4]Department of Chemistry, University of North Carolina, Chapel Hill, North Carolina 27599, USA

[5]Luxembourg Centre for Systems Biomedicine (LCSB), University of Luxembourg, 6 Avenue du Swing, 4367, Belvaux, Luxembourg

* Correspondence:

Ivan Rusyn (irusyn@tamu.edu), Erin S. Baker (erinmsb@unc.edu)

Instrument Settings. The listed instrument settings were used for the IMS-MS analysis of the chemical standards used in this study.

ESI Ionization Settings:
Gas temperature: 325 C
Drying Gas: 5 l/min
Nebulizer: 20 psi
Sheath Gas Temperature: 275 C
Sheath Gas Flow: 10 l/min
Fragmentor: 400 V
Oct 1 RF Vpp: 750 V

APCI Ionization Settings:
Gas temperature: 325 C
Vaporizer: 300 C
Drying Gas: 5 l/min
Nebulizer: 20 psi
VCap: 3500 V
Corona+: 4 μA
Fragmentor: 400 V
Oct 1 RF Vpp: 750 V

IMS Acquisition Settings (All Modes):
Frame Rate: 0.9 frames/ sec
IM Transient Rate: 18 IM transients/ frame
Max Drift Time: 60 ms
TOF Transient Rate: 600 transients/ IM transients
Trap Fill Time: 20000 μs
Trap Release Time: 100 μs

Positive Ion Mode Parameters:
IM Front Funnel – High Pressure Funnel Delta: 150 V
IM Front Funnel – High Pressure Funnel RF: 150 V
IM Front Funnel – Trap Funnel Delta: 180 V
IM Front Funnel – Trap Funnel RF: 150 V
IM Front Funnel – Trap Funnel Exit: 10 V
IM Trap – Trap Entrance Grid Low: 98 V
IM Trap – Trap Entrance Grid Delta: 10 V
IM Trap – Trap Entrance: 91 V
IM Trap – Trap Exit: 90 V
IM Trap – Trap Exit Grid 1 Low: 88.3 V
IM Trap – Trap Exit Grid 1 Delta: 4 V
IM Trap – Trap Exit Grid 2 Low: 87.3 V

IM Trap – Trap Exit Grid 2 Delta: 8.5 V
IM Drift Tube – Drift Tube Entrance Voltage: 1574 V
IM Drift Tube – Drift Tube Exit Voltage: 224 V
IM Rear Funnel – Rear Funnel Entrance: 217.5 V
IM Rear Funnel – Rear Funnel RF: 150 V
IM Rear Funnel – Rear Funnel Exit: 45 V
IM Rear Funnel – IM Hex Delta: -8 V
IM Rear Funnel – IM Hex RF: 600 V
IM Rear Funnel – IM Hex Entrance: 41 V
IM – IM Hex Delta Delta: 0 V
IM – Collision Cell Delta Delta: 0 V
IM – IBC Delta Delta: 0 V

Negative Ion Mode Parameters:
IM Front Funnel – High Pressure Funnel Delta: -150 V
IM Front Funnel – High Pressure Funnel RF: -150 V
IM Front Funnel – Trap Funnel Delta: -180 V
IM Front Funnel – Trap Funnel RF: -150 V
IM Front Funnel – Trap Funnel Exit: -10 V
IM Trap – Trap Entrance Grid Low: -98 V
IM Trap – Trap Entrance Grid Delta: -10 V
IM Trap – Trap Entrance: -91 V
IM Trap – Trap Exit: -90 V
IM Trap – Trap Exit Grid 1 Low: -88.1 V
IM Trap – Trap Exit Grid 1 Delta: -5 V
IM Trap – Trap Exit Grid 2 Low: -85.8 V
IM Trap – Trap Exit Grid 2 Delta: -10.5 V
IM Drift Tube – Drift Tube Entrance Voltage: -1574 V
IM Drift Tube – Drift Tube Exit Voltage: -224 V
IM Rear Funnel – Rear Funnel Entrance: -217.5 V
IM Rear Funnel – Rear Funnel RF: -150 V
IM Rear Funnel – Rear Funnel Exit: -45 V
IM Rear Funnel – IM Hex Delta: 8 V
IM Rear Funnel – IM Hex RF: -600 V
IM Rear Funnel – IM Hex Entrance: -41 V
IM – IM Hex Delta Delta: 0 V
IM – Collision Cell Delta Delta: 0 V
IM – IBC Delta Delta: 0 V
