# Supplementary material for: Reference library for suspect screening of environmental toxicants using ion mobility spectrometry-mass spectrometry
Source: Commun Chem. 2025 Aug 1;8:224. doi: 10.1038/s42004-025-01619-7 (PMC12317010; doi:10.1038/s42004-025-01619-7)
Supplement: Supplementary file 2 — Description of Additional Supplementary files [file 42004_2025_1619_MOESM2_ESM.pdf]

Description of Additional Supplementary files

File name: Supplementary Data 1

Description: Supplementary Tables (Tables S1-S13) have been supplied within the Supplementary Data file.
